# *Arabidopsis MYB24* Regulates Jasmonate-Mediated Stamen Development

**DOI:** 10.3389/fpls.2017.01525

**Published:** 2017-09-05

**Authors:** Huang Huang, Hua Gao, Bei Liu, Tiancong Qi, Jianhua Tong, Langtao Xiao, Daoxin Xie, Susheng Song

**Affiliations:** ^1^School of Life Sciences, Tsinghua University Beijing, China; ^2^College of Biological Science and Engineering, Beijing University of Agriculture Beijing, China; ^3^Beijing Key Laboratory of Plant Gene Resources and Biotechnology for Carbon Reduction and Environmental Improvement, College of Life Sciences, Capital Normal University Beijing, China; ^4^College of Bioscience and Biotechnology, Southern Regional Collaborative Innovation Center for Grain and Oil Crops in China, Hunan Agricultural University Changsha, China

**Keywords:** fertility, interaction, JAZs, MYB24, OPR3, stamen

## Abstract

The phytohormone jasmonates (JAs) regulate various defense responses and diverse developmental processes including stamen development and fertility. Previous studies showed that JA induces CORONATINE INSENSITIVE 1-mediated degradation of JA ZIM-domain (JAZ) proteins, and activates the MYB transcription factors (such as MYB21 and MYB24) to regulate stamen development. In this study, we further uncover the mechanism underlying how MYB24 interacts with JAZs to control JA-regulated stamen development. We show that N-terminus of MYB21/24 interacts with 10 out of 12 JAZ proteins while both N-terminus and C-terminus of MYB24 are involved in dimerization of MYB21 and MYB24. Interestingly, male sterility of the JA-deficient mutant *opr3* can be rescued by suitable level of the *MYB24* overexpression but not by excessive high level of *MYB24*. Surprisingly, overexpression of *MYB24NT*, but not *MYB24CT*, could cause male sterility. These results provide new insights on MYB factors in JA-regulated stamen development.

## Introduction

Jasmonates (JAs), a class of lipid-derived phytohormones (Browse, 2009; Wasternack and Song, 2017), are crucial players in various aspects of plant developmental processes (Huang et al., 2017), including root growth (Fernandez-Calvo et al., 2011; Lischweski et al., 2015), stamen development (Mandaokar et al., 2006; Mandaokar and Browse, 2009; Song et al., 2011; Reeves et al., 2012; Song et al., 2013b), flowering (Zhai et al., 2015), trichome initiation (Qi et al., 2011), and leaf senescence (Qi et al., 2015b); they also mediate plant abiotic stress tolerance and defenses against herbivores and necrotrophic pathogens (Howe and Jander, 2008; Campos et al., 2014; Kazan, 2015; Goossens et al., 2016).

In response to developmental signals or environmental cue-triggered JA biosynthesis, the JA receptor CORONATINE INSENSITIVE 1 (COI1) (Xie et al., 1998; Yan et al., 2009) perceives bioactive molecules of JA (Fonseca et al., 2009; Yan et al., 2016) to recruit JA ZIM-domain (JAZ) proteins for ubiquitination and subsequent degradation via the 26S-proteasome (Chini et al., 2007; Thines et al., 2007; Yan et al., 2007), thereby de-repressing JAZ-inhibited transcription factors, such as MYC2/3/4/5 (Cheng et al., 2011; Fernandez-Calvo et al., 2011; Niu et al., 2011; Song et al., 2014a; Figueroa and Browse, 2015; Qi et al., 2015a; Gimenez-Ibanez et al., 2017; Major et al., 2017), MYB21/24 (Song et al., 2011), IIId bHLH factors (Nakata et al., 2013; Nakata and Ohme-Takagi, 2013; Song et al., 2013a), and TTG1/bHLH/MYB complexes (Qi et al., 2011) to modulate distinct JA responses.

Jasmonate-deficient mutants (e.g., *aos* and *opr3*), the JA receptor mutant *coi1-1*, and JAZ dominant-negative transgenic plants (*JAZ1Δ3A*) are all male sterile with defects in filament elongation, anther dehiscence, and pollen maturation (Xie et al., 1998; Stintzi and Browse, 2000; Park et al., 2002; Thines et al., 2007). The R2R3–MYB transcription factors MYB21 and MYB24 associate with IIIe bHLH factors (MYC2, MYC3, MYC4, and MYC5) to form MYB–MYC complexes, and interact with JAZs to mediate late stamen development (Qi et al., 2015a).

We previously showed that JAZ1/8/11 interact with MYB21/MYB24 in yeast and plants (Song et al., 2011). In this study, we further showed that MYB21 and MYB24 interact with most JAZs via their N-terminal R2R3 domains, and both the N-terminus and C-terminus of MYB24 mediate the dimeric interactions of MYB21 and MYB24. Proper overexpression of *MYB24* partially restores male fertility of *opr3*. Overexpression of N-terminus of *MYB24*, but not C-terminus, causes male sterility in wild-type. Furthermore, young flower buds from *myb21 myb24 myb57* accumulate more jasmonic acid than that of wild-type.

## Materials and Methods

### Plant Materials and Growth Conditions

The *Arabidopsis* mutants *opr3* (Stintzi and Browse, 2000) and *myb21 myb24 myb57* (Cheng et al., 2009) were described previously. *Arabidopsis thaliana* seeds were disinfected, germinated on Murashige and Skoog (MS) medium, stored at 4°C for 3 days, and transferred to a growth room for another 7 days before being transferred to soil under a 16 h (22–24°C)/8 h (17–19°C) light/dark photoperiod. *Nicotiana benthamiana* seeds were sown in soil and grown under a 16 h (26°C)/8 h (22°C) light/dark photoperiod.

### Yeast Two-Hybrid Assay

The full lengths of coding regions of *MYB21, MYB24*, 12 *JAZs*, and truncated domains of *MYB24* were individually fused with the activation domain (AD) in pB42AD, or the DNA binding domain (BD) in pLexA. The primer pairs used for vector construction are listed in Supplementary Table 1. Yeast transformation and protein–protein interaction assays were performed according to the Matchmaker LexA Two-Hybrid System (Clontech) as described previously (Song et al., 2011).

### Firefly Luciferase (LUC) Complementation Imaging (LCI) Assay

*JAZ5, MYB21, MYB24, MYB24NT*, and *MYB24CT* were individually inserted into pCAMBIA-nLUC or pCAMBIA-cLUC for fusion with the N-terminal half of LUC (nLUC) or C-terminal half of LUC (cLUC). The primers used to construct the LCI vector are listed in Supplementary Table 1. *Agrobacterium tumefaciens* cells containing the indicated plasmids were co-infiltrated into *N. benthamiana* leaves and LUC activity was detected as described previously (Song et al., 2011).

## Generation of Transgenic Plants

To obtain transgenic plants overexpressing *MYB24*, the coding sequence of *MYB24* was amplified and cloned using *Xba*I and *Sac*I into pCAMBIA1301 under the control of the *CaMV 35S* promoter. The construct was transformed into *OPR3*/*opr3* heterozygous plants using the *Agrobacterium*-mediated floral dip method. The primers used for vector construction are presented in Supplementary Table 1. *pCAMBIA-MYB24NT-nLUC* and *pCAMBIA-MYB24CT-nLUC* were transformed into *Arabidopsis* Col-0 wild-type to generate *MYB24NT*- and *MYB24CT*-overexpressing plants.

### Quantitative Real-time PCR

Young flower buds of plants were collected for total RNA extraction and subsequent reverse transcription. Quantitative real-time PCR was performed with RealMasterMix (SYBR Green I; Takara; Bio Inc., Otsu, Japan) using the ABI 7500 Real-Time PCR System (Applied Biosystems, Foster City, CA, United States), and *ACTIN8* was used as an internal control. The primers used are listed in Supplementary Table 1.

### Flower Phenotype Analysis and Pollen Germination Assay

For flower phenotype analysis, flowers of each genotype at stage 13 were photographed under a microscope. Pollen germination assay was conducted as described previously (Song et al., 2011). Pollen grains were germinated on pollen germination medium [1 mM MgSO_4_, 5 mM CaCl_2_, 5 mM KCl, 10% (w/v) sucrose, 0.01% boric acid, and 1.5% agar at pH 7.5], incubated for 10 h at 22°C in the dark, and observed under a microscope.

### Jasmonic Acid Measurement

Five hundred milligrams of young flower buds before floral stage 13 from 5-week-old wild-type and the *myb21 myb24 myb57* mutant was harvested, and jasmonic acid was extracted and quantified as described previously using a liquid chromatography–tandem mass spectrometry system (Cheng et al., 2009).

### Accession Numbers

The *Arabidopsis* Genome Initiative numbers for genes mentioned in this article are as follows: JAZ1 (At1g19180), JAZ2 (At1g74950), JAZ3 (At3g17860), JAZ4 (At1g48500), JAZ5 (At1g17380), JAZ6 (At1g72450), JAZ7 (At2g34600), JAZ8 (At1g30135), JAZ9 (At1g70700), JAZ10 (At5g13220), JAZ11 (At3g43440), JAZ12 (At5g20900), MYB21 (At3g27810), MYB24 (At5g40350), MYB57 (At3g01530), OPR3 (AT2G06050), and ACTIN8 (At1g49240).

## Results

### MYB21 and MYB24 Interact with Multiple JAZs via Their N-terminus

**FIGURE 1:**
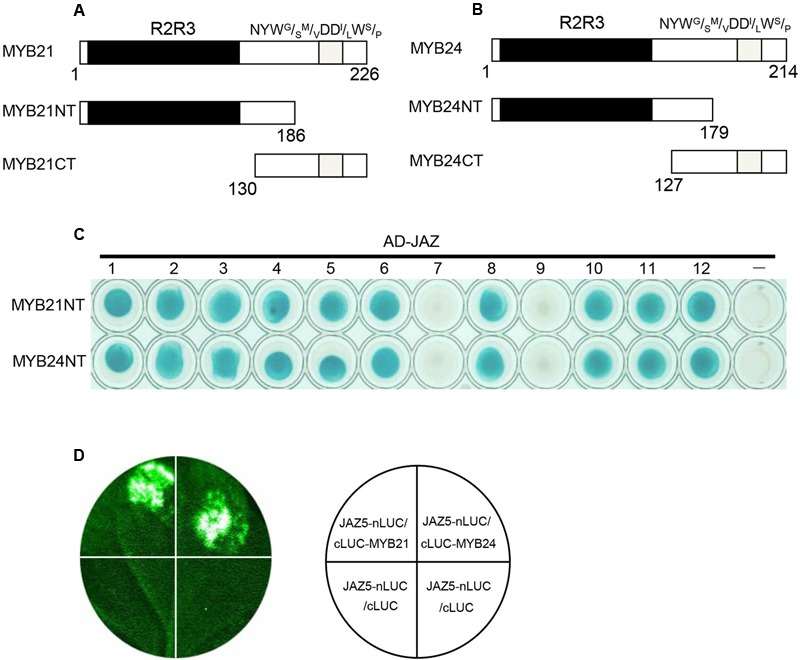
MYB21 and MYB24 interact with JAZ proteins via their N-terminus. **(A,B)** Schematic diagram of MYB21 **(A)** and MYB24 **(B)** with the R2R3 domain (black) and NYW^G^/_S_^M^/_V_DD^l^/_L_W^S^/_P_ motif (gray). **(C)** Y2H assays to show interactions between MYB21NT/MYB24NT and JAZs. MYB21NT and MYB24NT were fused with DNA binding domain (BD) in pLexA, and JAZs were fused with activation domain (AD) in pB42AD, respectively. **(D)** JAZ5 interacts with MYB21 and MYB24 in LCI assay. JAZ5 was fused with N-terminal fragment of LUC (nLUC), while MYB21/MYB24 were fused with C-terminal fragment of LUC (cLUC), respectively. LUC signals were detected 60 h after co-infiltration of the indicated constructs into *N. benthamiana* leaves.

MYB21 and MYB24 were divided into MYB21NT and MYB24NT harboring the R2R3 DNA BD, and MYB21CT and MYB24CT containing the C-terminal NYW^G^/_S_^M^/_V_DD^I^/_L_W^S^/_P_ transcriptional activation motif (**Figures 1A,B**). Binding domain-fused MYB21CT and MYB24CT exhibited auto-activation, while MYB21NT and MYB24NT did not. Binding domain-fused MYB21NT and MYB24NT were used to detect interactions with AD-fused JAZ proteins. The results (**Figure 1C**) showed that MYB21NT and MYB24NT interact with JAZ1, JAZ2, JAZ3, JAZ4, JAZ5, JAZ6, JAZ8, JAZ10, JAZ11, and JAZ12, but not with JAZ7 or JAZ9, demonstrating that MYB21 and MYB24 interact with multiple JAZs through their N-terminus.

We next performed a firefly LCI assay to test the interactions of JAZ5 with MYB21/24 in plant. JAZ5 was fused with nLUC, while MYB21 and MYB24 were fused with cLUC. The results showed that co-infiltration of JAZ5-nLUC/cLUC-MYB21 or JAZ5-nLUC/cLUC-MYB24 in *N. benthamiana* leaves resulted in strong LUC signals while the negative control did not (**Figure 1D**), suggesting that MYB21 and MYB24 interact with JAZ5 in plant.

### *MYB24* Overexpression Restores Stamen Development in *opr3*

**FIGURE 2:**
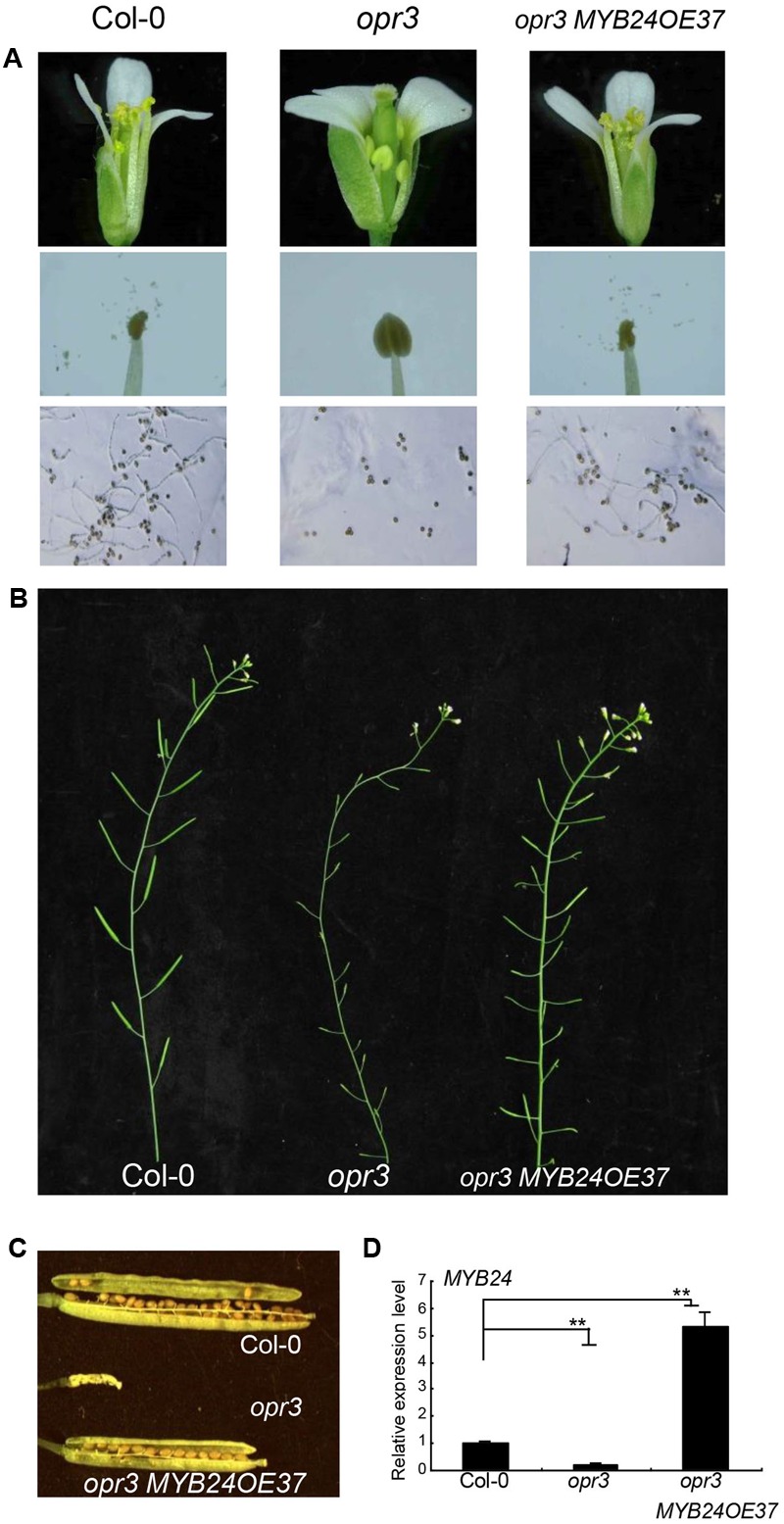
Overexpression of *MYB24* partially restores the stamen development and fertility of *opr3*. **(A–C)** Comparison of flowers, stamens, and pollen germination **(A)** at floral stage 13, main inflorescence **(B)**, and seed setting **(C)** in Col-0, *opr3*, and *opr3 MYB24OE37*. *MYB24* overexpression partially restored the fertility of *opr3* **(A–C)**. **(D)** Quantitative real-time PCR analysis of *MYB24* expression in young flower buds of Col-0, *opr3*, and *opr3 MYB24OE37*. *ACTIN8* was used as the internal control. Data are means (±SE) of three biological replicates. Asterisks represent Student’s *t*-test significance between pairs indicated with brackets (^∗∗^*p* < 0.01).

We next examined whether *MYB24* overexpression could escape from inhibition by multiple JAZs to rescue stamen development and fertility in the JA-deficient mutant *opr3*. As shown in **Figure 2A**, *opr3* exhibited unelongated filaments, indehiscent anthers, and inviable pollen grains at floral stage 13. *MYB24* expression decreased in young *opr3* flower buds (**Figure 2D**). *MYB24* overexpression in *opr3* (*opr3 MYB24OE37*, with fivefold to sixfold of wild-type level) restored filament elongation, anther dehiscence, and pollen viability (**Figure 2A**). Further, the *opr3 MYB24OE37* plants were able to set seeds (**Figures 2B,C**). These results suggest that overexpression of *MYB24* could partially restore stamen development and fertility in *opr3*.

### Excess Expression of *MYB24* Cannot Rescue the Fertility of the *opr3* Mutant

**FIGURE 3:**
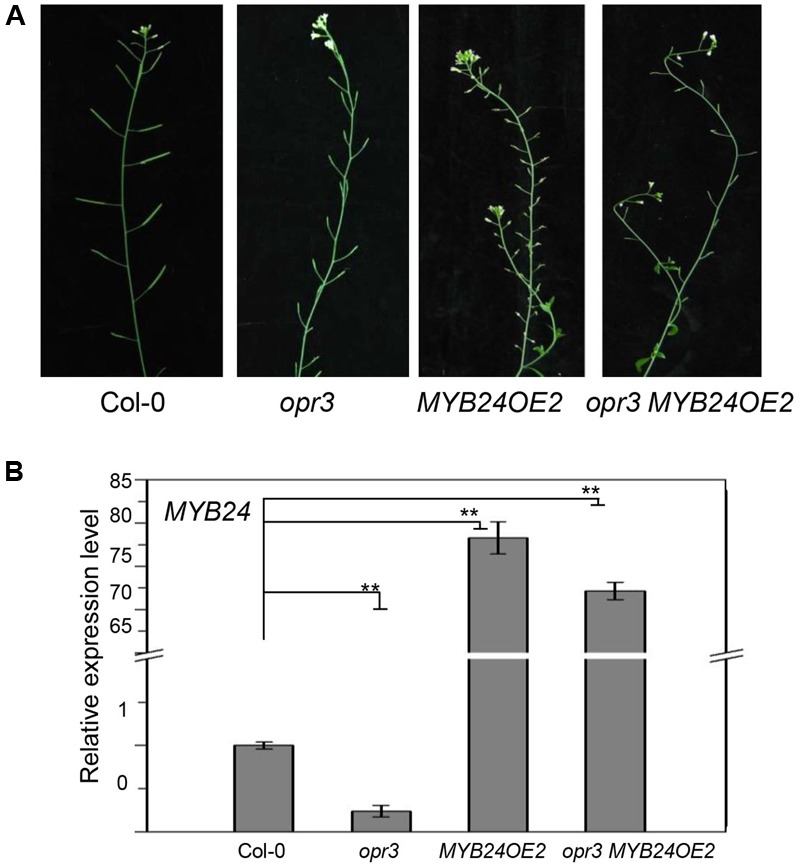
Excess expression of *MYB24* fails to rescue the fertility of *opr3*. **(A)** Main inflorescences of Col-0, *opr3, MYB24OE2*, and *opr3 MYB24OE2.* **(B)** Quantitative real-time PCR analysis of *MYB24* expression in young flower buds. *ACTIN8* was used as the internal control. Data are means (±SE) of three biological replicates. Asterisks represent Student’s *t*-test significance between pairs indicated with brackets (^∗∗^*p* < 0.01).

Previous studies showed that strong *MYB24* overexpression inhibits stamen development (Yang et al., 2007; Song et al., 2011). We therefore tested whether excess expression of *MYB24* could restore fertility in *opr3*. As shown in **Figure 3**, transgenic plants that expressed excessive amounts of *MYB24* (∼75-fold of the wild-type level) were male sterile. Further, transgenic *opr3* plants that expressed excessive amounts of *MYB24* (∼70-fold of the wild-type level) were still male sterile, suggesting that excess expression of *MYB24* could not restore stamen development and fertility in *opr3* plants.

### The N-terminus and C-terminus of MYB24 Are Involved in Dimeric Interactions

**FIGURE 4:**
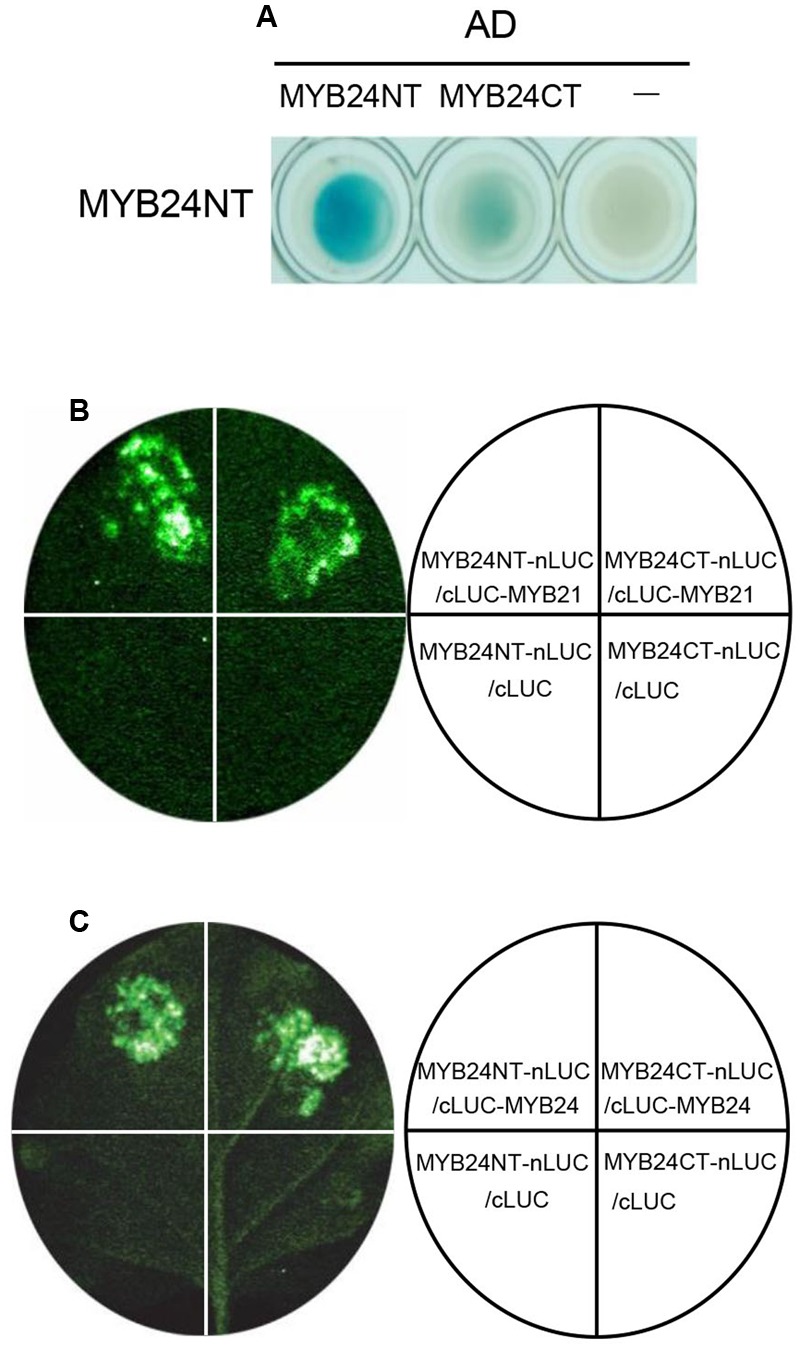
MYB24NT and MYB24CT mediate dimeric interactions. **(A)** Y2H assays to show the interactions of BD-fused MYB24NT with AD-fused MYB24NT and MYB24CT. **(B,C)** LCI assays show that both MYB24NT and MYB24CT interact with MYB21 **(B)** and MYB24 **(C)**. MYB24NT and MYB24CT were fused with nLUC, while MYB21/MYB24 were fused with cLUC, respectively. LUC signals were collected 60 h after co-infiltration.

We next investigated the dimeric interactions of MYB21 and MYB24 in detail. As shown in **Figure 4A**, Y2H analysis showed that BD-fused MYB24NT interacted strongly with AD-fused MYB24NT, and weakly with MYB24CT. Further, LCI assay exhibited that the co-expression of MYB24NT-nLUC/cLUC-MYB21, MYB24CT-nLUC/cLUC-MYB21, MYB24NT-nLUC/cLUC-MYB24, and MYB24CT-nLUC/cLUC-MYB24 resulted in strong LUC activity, while the negative controls did not (**Figures 4B,C**). These results demonstrate that both N-terminus and C-terminus of MYB24 are involved in dimeric interactions of MYB21 and MYB24.

### *MYB24NT* Overexpression Causes Male Sterility

**FIGURE 5:**
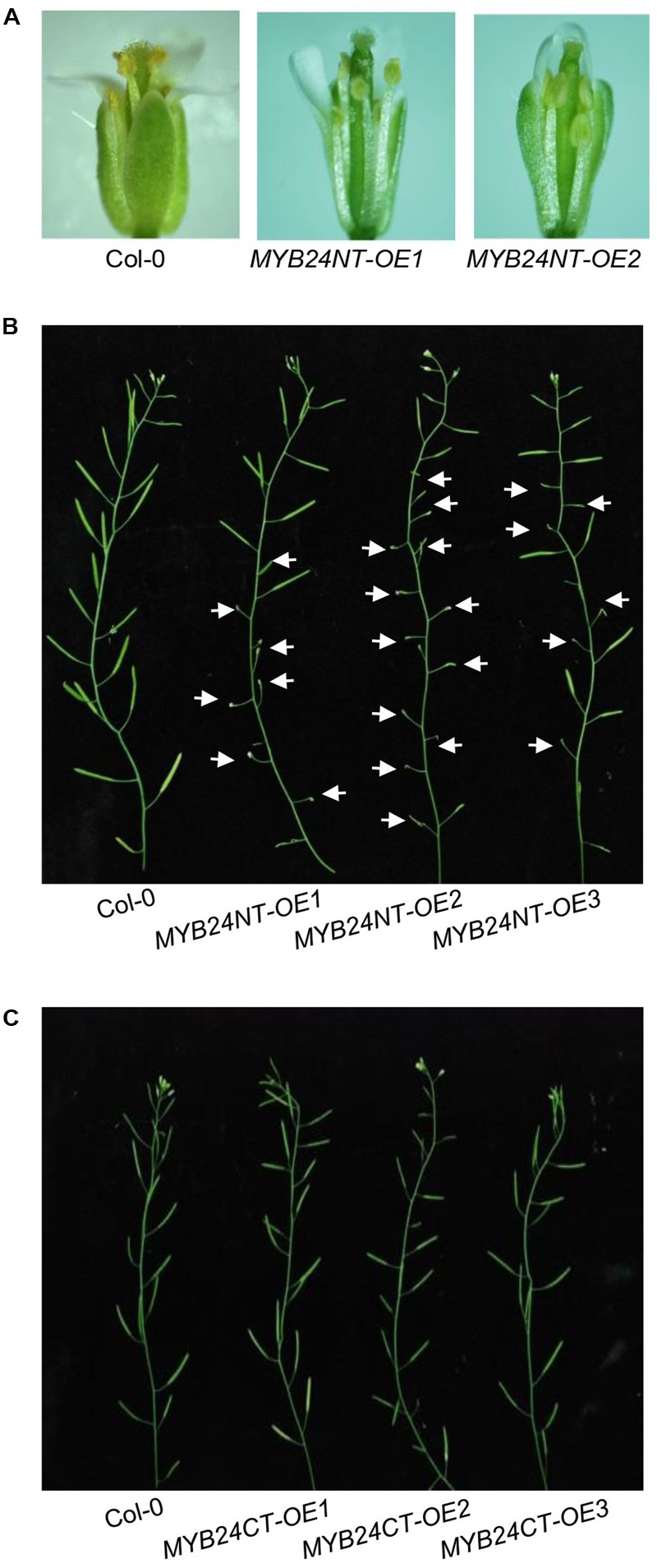
Excess expression of *MYB24NT* causes male sterility. **(A)** Comparison of flowers at stage 13 from Col-0 and two *MYB24NT* overexpression lines (*MYB24NT-OE1* and *MYB24NT-OE2*). **(B)** Main inflorescences of Col-0, *MYB24NT-OE1, MYB24NT-OE2*, and *MYB24NT-OE3.* White arrows indicate sterile siliques. **(C)** Main inflorescences of Col-0, *MYB24CT-OE1, MYB24CT-OE2*, and *MYB24CT-OE3*.

We next examined whether the overexpression of *MYB24NT* and *MYB24CT* could dominantly repress stamen development and male fertility. *MYB24NT* overexpression (∼20–40-fold of the wild-type level) inhibited stamen development, including filament elongation, anther dehiscence, and male fertility (**Figures 5A,B** and Supplementary Figure 1A), while all *MYB24CT* transgenic lines showed no obvious influence on male fertility (**Figure 5C** and Supplementary Figure 1B). These data indicate that overexpression of N-terminus of *MYB24* could dominantly repress stamen development and fertility.

### JA Concentration Increased in Young Flower Buds of *myb21 myb24 myb57*

**FIGURE 6:**
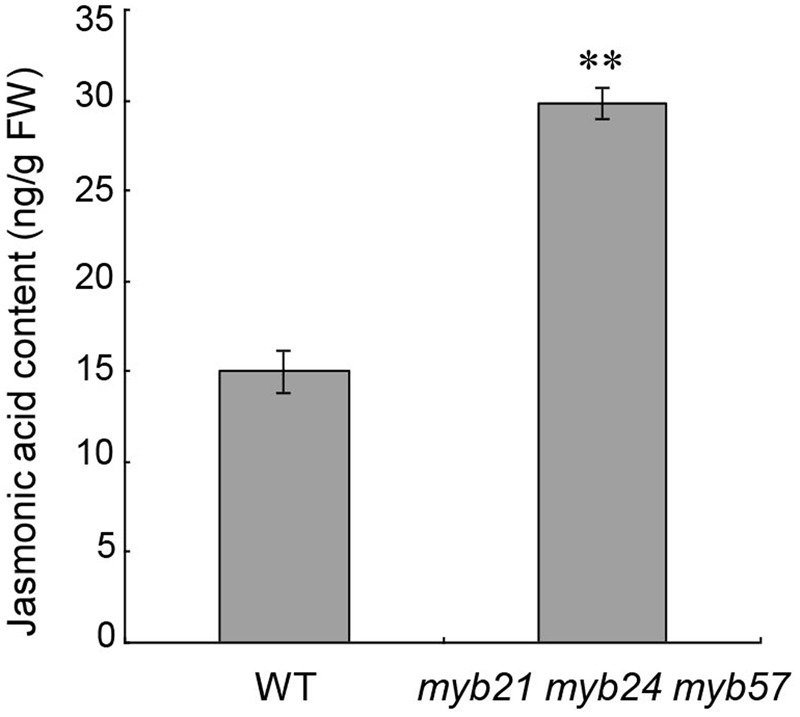
Jasmonic acid contents of young flower buds from wild-type and *myb21 myb24 myb57*. Jasmonic acid contents of young flower buds before floral stage 13 from wild-type and *myb21 myb24 myb57* plants. Data are means (±SE) of three biological replicates. Asterisks represent Student’s *t*-test significance compared with Col-0 wild-type (^∗∗^*p* < 0.01).

*MYB21, MYB24*, and *MYB57* are all responsive to JA (**Figure 1**; Mandaokar et al., 2006; Cheng et al., 2009; Mandaokar and Browse, 2009). We thus tested whether *MYB21, MYB24*, and *MYB57* in turn regulate JA biosynthesis in young flower buds (before floral stage 13). The jasmonic acid content of young *myb21 myb24 myb57* mutant flower buds was approximately twofold of the wild-type level (**Figure 6**), suggesting that *MYB21, MYB24*, and *MYB57* negatively regulate JA biosynthesis as part of a negative feedback loop.

## Discussion

Jasmonate ZIM-domain proteins serve as repressors that target specific transcription factors and control their downstream pathways to modulate distinct JA responses for coordinated regulation of development, growth, and defense (Song et al., 2014b; Chini et al., 2016; Gimenez-Ibanez et al., 2017; Major et al., 2017). Our previous study showed that JAZ1, JAZ8, and JAZ11 interact with MYB21 and MYB24 (Song et al., 2011). In this study, we further showed that MYB21 and MYB24 act through N-terminus to interact with 10 out of 12 JAZs (**Figure 1**), suggesting that most JAZs may act through interfering the DNA binding function of MYB21/24 to attenuate their function in regulating stamen development. Excess expression of *MYB24* is unable to restore the stamen development of *opr3*, whereas suitable overexpression of *MYB24* could recover stamen development and male fertility (**Figures 2, 3**). Exploring the downstream pathways of MYB24 would help to understand that the suitable *MYB24* expression level is essential for proper stamen development.

Both the N-terminal DNA BD and C-terminal transcriptional activation motif mediate dimerization of MYB21 and MYB24 (**Figure 4**). Determination of crystal structure of MYB21/24 will help to further elucidate the interaction. Interestingly, overexpression of N-terminus of *MYB24*, but not the C-terminus of *MYB24*, attenuates stamen development and male fertility (**Figure 5**). *MYB24NT-OE2* with the highest expression level of *MYB24NT* confers the most severe male sterility (**Figures 5A,B** and Supplementary Figure 1A), suggesting that the expression level of *MYB24NT* is correlated with male sterility. It remains to study whether MYB24NT affects the dimeric interaction of MYBs to affect stamen development.

We also found that young flower buds of *myb21 myb24 myb57* accumulated more jasmonic acid (**Figure 6**), suggesting that MYBs negatively regulate JA biosynthesis to attenuate JA-induced expression of *MYBs* and to elaborately regulate stamen development, and that the restored fertility in *opr3* (Stintzi and Browse, 2000; Chehab et al., 2011) by suitable *MYB24* overexpression (**Figure 2**) is not due to recovery of JA biosynthesis. It would be useful for understanding the MYB21/24 module in stamen development if the links between MYB21/24 and JA biosynthetic genes are elucidated.

## Author Contributions

DX and SS designed the study; HH, HG, BL, TQ, JT, LX, and SS performed the experiments; HH, HG, BL, TQ, JT, LX, and SS analyzed the data; and HH, HG, and SS wrote the manuscript.

## Conflict of Interest Statement

The authors declare that the research was conducted in the absence of any commercial or financial relationships that could be construed as a potential conflict of interest.
