# Supplementary material for: Arabidopsis MYB24 Regulates Jasmonate-Mediated Stamen Development
Source: Front Plant Sci. 2017 Sep 5;8:1525. doi: 10.3389/fpls.2017.01525 (PMC5591944; doi:10.3389/fpls.2017.01525)
Supplement: Supplementary file 1 [file Table_1.DOC]

**Supplemental Table 1. Primers used for vector construction and quantitative real-time PCR analysis.**

| JAZ1 AD | Forward | cccgaattcatgtcgagttctatggaatg |
| --- | --- | --- |
| JAZ1 AD | Reverse | aaaagtcgactcatatttcagctgctaaacc |
| JAZ2 AD | Forward | cccgaattcatgtcgagtttttctgccgagtg |
| JAZ2 AD | Reverse | accgctcgagttaccgtgaactgagccaagctg |
| JAZ3 AD | Forward | ggggaattcatggagagagattttctcgggttg |
| JAZ3 AD | Reverse | cccgctcgagttaggttgcagagctgagagaag |
| JAZ4 AD | Forward | ggggaattc atggagagagattttctcgggctg |
| JAZ4 AD | Reverse | cccgctcgagttagtgcagatgatgagctggag |
| JAZ5 AD | Forward | cccgaattcatgtcgtcgagcaatgaaaatgc |
| JAZ5 AD | Reverse | cccgctcgagctatagccttagatcgagatc |
| JAZ6 AD | Forward | ggggaattcatgtcaacgggacaagcgccggag |
| JAZ6 AD | Reverse | cccgctcgagctaaagcttgagttcaaggtt |
| JAZ7 AD | Forward | cccgaattcatgatcatcatcatcaaaaactg |
| JAZ7 AD | Reverse | accgctcgagctatcggtaacggtggtaagg |
| JAZ8 AD | Forward | ccccccatggatgaagctacagcaaaattgtg |
| JAZ8 AD | Reverse | gggctcgagttatcgtcgtgaatggtacggtg |
| JAZ9 AD | Forward | ggggaattc atggaaagagattttctgggtttg |
| JAZ9 AD | Reverse | ccgctcgagttatgtaggagaagtagaagag |
| JAZ10 AD | Forward | ggggaattcatgtcgaaagctaccatagaactcg |
| JAZ10 AD | Reverse | acgcgtcgac ttaggccgatgtcggatagtaag |
| JAZ11 AD | Forward | ggggaattcatggctgaggtaaacggagatttc |
| JAZ11 AD | Reverse | aaaagtcgactcatgtcacaatggggctgg |
| JAZ12 AD | Forward | cccggatccatgactaaggtgaaagatgagcc |
| JAZ12 AD | Reverse | acgcgtcgacctaagcagttggaaattcctcc |
| MYB21NT BD | Forward | cgccaattgatggagaaaagaggaggaggaag |
| MYB21NT BD | Reverse | cccgctcgagtcacgaatagttaccatagttg |
| MYB24NT BD/AD | Forward | ggggaattcatggagaaaagagaaagtagtg |
| MYB24NT BD/AD | Reverse | ccgctcgagtcaataattaccataattaagc |
| MYB24CT AD | Forward | ggggaattcacgacgaccgttggatcacaaa |
| MYB24CT AD | Reverse | cccgctcgagttaattaccattatatatattc |
| JAZ5 nLUC-F | Forward | cccgagctcatgtcgtcgagcaatgaaaatg |
| JAZ5 nLUC-R | Reverse | acgcgtcgactagccttagatcgagatctttc |
| MYB21cLUC | Forward | cgcggatccatggagaaaagaggaggaggaag |
| MYB21cLUC | Reverse | acgcgtcgactcaattaccattcaataaatgc |
| MYB24cLUC | Forward | cgcggatccatggagaaaagagaaagtagtg |
| MYB24cLUC | Reverse | acgcgtcgacttaattaccattatatatattc |
| MYB24NT nLUC-F | Forward | cccgagctcatggagaaaagagaaagtagtg |
| MYB24NT nLUC-R | Reverse | acgcgtcgacataattaccataattaagctgc |
| MYB24CT nLUC-F | Forward | cccgagctcatgacgacgaccgttggatcacaaagc |
| MYB24CT nLUC-R | Reverse | acgcgtcgacattaccattatatatattcatg |
| MYB24 qrt-PCR | Forward | tggaactctctcgccaaatc |
| MYB24 qrt-PCR | Reverse | gcacatcaggtcggaggtag |
| MYB24NT qrt-PCR | Forward | tggaactctctcgccaaatc |
| MYB24NT qrt-PCR | Reverse | gcacatcaggtcggaggtag |
| MYB24CT qrt-PCR | Forward | ggtaattatgtgcctgaatccg |
| MYB24CT qrt-PCR | Reverse | ggccaaagatcatcgacgct |
| 35S*MYB24*OE-F | Forward | agctcgagatggagaaaagagaaagtagtg |
| 35S*MYB24*OE-R | Reverse | atcgagctcttaattaccattatatatattcatg |
| Actin8 | Forward | tcagcactttccagcagatg |
| Actin8 | Reverse | ctgtggacaatgcctggac |
