# Supplementary material for: Arabidopsis MYB24 Regulates Jasmonate-Mediated Stamen Development
Source: Front Plant Sci. 2017 Sep 5;8:1525. doi: 10.3389/fpls.2017.01525 (PMC5591944; doi:10.3389/fpls.2017.01525)
Supplement: Supplementary file 2 [file Image_1.PDF]

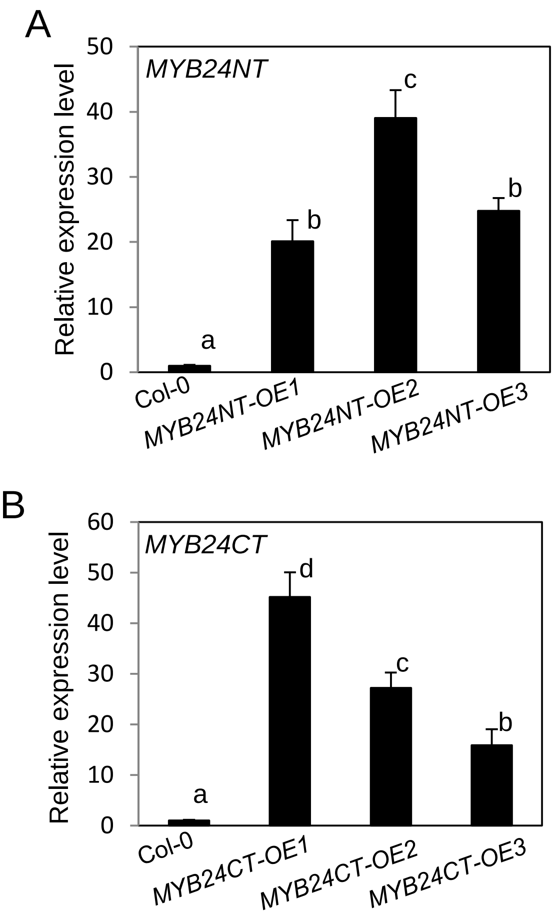

Supplementary Figure 1. Quantitative real-time PCR analysis of expression levels of *MYB24NT* and *MY24CT* in transgenic plants.

(A) and (B) Quantitative real-time PCR analysis of *MYB24NT* (A) or *MYB24CT* expression (B) in young flower buds from Col-0, and their transgenic plants *MYB24NT-OE1*, *MYB24NT-OE2* and *MYB24NT-OE3* (A), or *MYB24CT-OE1*, *MYB24CT-OE2* and *MYB24CT-OE3* (B). *ACTIN8* was used as the internal control. Data are means (±SE) of three biological replicates. Lowercase letters indicate significant differences by a one-way ANOVA ($P < 0.05$).
